# Who does it first? The uptake of medical innovations in the performance of thrombolysis on ischemic stroke patients in Germany: a study based on hospital quality data

**DOI:** 10.1186/s13012-014-0196-7

**Published:** 2015-01-13

**Authors:** Nadine Scholten, Holger Pfaff, Helmar C Lehmann, Gereon R Fink, Ute Karbach

**Affiliations:** IMVR—Institute for Medical Sociology, Health Services Research, and Rehabilitation Science, University of Cologne, Cologne, Germany; Department of Neurology, University Hospital Cologne, Cologne, Germany; Cognitive Neuroscience, Institute of Neuroscience and Medicine (INM-3), Research Center Jülich, Jülich, Germany

**Keywords:** Ischemic stroke, Thrombolysis, Guideline, Implementation, Neurology

## Abstract

**Background:**

Since 2000, systemic thrombolysis has been the only approved curative and causal treatment for acute ischemic stroke. In 2009, the guidelines of the German Society for Neurology were updated and the therapeutic window for performing thrombolysis was extended. The implementation of new therapies is influenced by many factors. We analyzed the factors at the organizational level that influence the implementation of thrombolysis in stroke patients.

**Methods:**

The data published by the majority of German hospitals in their structured quality reports was assessed. We calculated a regression model in order to measure the influence of hospital/department-level characteristics (e.g., teaching status, ownership, location, and number of hospital beds) on the implementation of thrombolysis in 2006 (this is the earliest point in time that can be analyzed on this data basis). In order to measure the effect of the guideline update in 2009 on the thrombolysis rate (TR) change between 2008 and 2010, we performed a Wilcoxon signed-rank test and utilized a regression model.

**Results:**

In 2006, 61.5% of a total of 286 neurology departments performed systemic thrombolysis to treat ischemic strokes. The influencing factors for the use of systemic thrombolysis in 2006 were the existence of a stroke unit (+) and a hospital size of between 500 and 1,000 beds (−). A significant increase of the mean departmental TR (thrombolysis rate) from 6.7% to 9.2% between 2008 and 2010 was observed after the guideline update in 2009. For the departments performing thrombolysis in 2008 and 2010, our analysis could not show any additional influencing factors on a structural level that would explain the TR rise during the period 2008–2010.

**Conclusions:**

Because ischemic stroke patients benefit from systemic thrombolysis, it is necessary to examine possible barriers at the organizational level that hinder the implementation. Our data shows that, organizational factors have an influence on the implementation of thrombolysis. However, the recent guideline update resulted in a TR rise that occurred at all hospitals, regardless of the measured structural conditions, as our analysis could not identify any structural factors that might have influenced the TR after the guideline update.

## Background

Strokes are the most frequent cause of death worldwide, after ischemic heart diseases [1]. Over the last decade, the global burden of stroke, measured by disability-adjusted life years (DALYs) has increased, with stroke being the third leading cause of health loss [2]. With about 243,000 hospitalizations in 2010, acute stroke is one of the most common diseases in Germany [3]. The incidence of stroke in Germany is similar to the incidence in the US and the other EU5 countries [4]. To date, systemic thrombolysis with recombinant tissue plasminogen activator (rtPA, alteplase) constitutes the only approved curative and causal therapy for treating acute ischemic stroke patients [5]. The most promising alternative approach is endovascular thrombectomy. Thus far, however, trials performed have failed to demonstrate equivalent or superior therapeutic results [6-8]. Therefore, even if intra-arterial treatment is considered, systemic thrombolysis should still be performed as a first-line treatment in all suitable patients, as systemic thrombolysis remains the only evidence-based therapeutic option for patients reaching a hospital within the therapeutic window [9]. RtPA was licensed for the treatment of acute ischemic stroke in Germany in 2000, 4 years after its approval by the US Food and Drug Administration (FDA) [10] and 2 years before the European Union granted a license in 2002 [11]. With this first approval, the use of rtPA was restricted to a limited time frame of 3 h after symptom onset. On the basis of subsequent studies [12,13], the guidelines of the German Society for Neurology [11], as well as several other national and international guidelines, were updated in 2009. The recommended therapeutic window for performing systemic thrombolysis was then extended from 3 to 4.5 h [14-16]. Because of this increased time window, more acute ischemic stroke patients should be able to receive systemic thrombolysis [17], and as a result, the thrombolysis rate (percentage of patients receiving thrombolysis, TR) would be expected to rise.

The function of a guideline is to provide recommendations for the optimal treatment scheme and to set treatment standards. Recommendations are based on the latest study results and are therefore evidence-based. Nevertheless, only a small percentage of these guidelines are implemented into clinical routines [18,19]. Especially with regard to the treatment of acute events like ischemic stroke, evidence-based guidelines, though widely disseminated, are poorly adhered to [20,21]. Nevertheless, a guideline promoting a new medical treatment regime represents an innovation that should be incorporated into a care provider’s actions and a hospital’s daily routines as quickly as possible. The implementation process starts with the decision to implement the therapeutic innovation, followed by its routine use [22]. The decision can be made by a senior organizational manager (in terms of organizational issues) or by the medical department heads where medical innovations are involved.

There are, however, many factors that influence the successful implementation of evidence-based health innovations. According to the conceptual framework used by Chaudoir et al., there are five different levels (the structural, organizational, patient, provider, and innovation levels) that influence the implementation outcome [23].

Hospital characteristics on the organizational-structural level—for example, teaching status, ownership, hospital size—have been found to have an impact on adherence to recommended care processes and evidence-based guidelines [24-26]. Another factor on the structural level that was found to have an influence on a hospital’s performance with respect to guideline-congruent treatment is the hospital’s location (rural vs. urban) [27,28]. For the implementation of clinical guidelines for the treatment of acute ischemic stroke patients, qualitative studies show that a health professional’s adherence to clinical guidelines is—at least to a great extent—influenced by the organizational-structural level [29,30]. Regarding the influence of teaching status on the performance of thrombolysis, a study from the Netherlands found that systemic thrombolysis was utilized far more often in an academic setting [30]. In addition, non-university hospitals in Sweden took 2–3 years longer to implement thrombolysis, with such hospitals not recording thrombolysis rates similar to university hospitals until 2008 [31]. With regard to a hospital’s location, rural hospitals in Australia seem to have the same thrombolysis rate as hospitals located in metropolitan regions [32].

A comparison of countries shows that the TR ranges from 1.4% in the UK (2008) [33] to 5% (2009) in the US [34], with 64% of US hospitals not applying any thrombolysis (2007) [35]. In 2010, the thrombolysis rate in Germany was 8.9% [3], whereby 38% of hospitals that treated ischemic stroke patients did not perform systemic thrombolysis [36]. Since systemic thrombolysis is the only approved curative and causal therapy for treating patients with an acute ischemic stroke, it is important to know what enables or prevents hospitals from implementing this therapy into their clinical routines. Thus, the aim of this study was to identify factors at the organizational level that facilitate early implementation of guidelines for treating acute ischemic stroke patients. The definition of ‘early implementation’ is based on the works of Rogers [37]. Because the implementation of a new technology occurs in different phases, the adoption of an innovation can be differentiated by its timing.

Our study aims to answer the following question: which factors at the organizational level—such as teaching status, experience in treating ischemic stroke patients, and ownership—have an impact upon a hospital department’s decision to implement systemic thrombolysis as a treatment for acute ischemic stroke patients early on. In addition to analyzing systemic thrombolysis implementation and the treatment situation of acute ischemic stroke patients in 2006, we explored whether the guideline update in 2009 led to changes in clinical routines, and we also examined which organizational issues influenced this process.

## Methods

Our study is based on data provided by German hospitals in their structured quality reports, which all German hospitals have been required to publish since 2005. The content and structure are determined by the Federal Joint Committee (G-BA) in Germany. The structured quality reports are published every 2 years and include structural as well as process data. All main diagnoses (ICD codes) and performed procedures are reported for each hospital department. The data provided can be used to develop a comprehensive picture of the health-care provision in Germany. Our analyses are based on data published in 2006, as this is the earliest possible data available. However, we also used data from 2008 and 2010. We included all hospitals that published a structured quality report and treated ischemic stroke patients (main diagnosis ICD code GM: I63) in a neurology department. The performance of thrombolysis and the existence of a stroke unit (i.e., a unit specialized in the treatment of acute stroke patients) were identified on the basis of documented Operationen- und Prozedurenschlüssel (OPS) codes, which are a national classification of health interventions in Germany, analogous to the International Classification of Procedures in Medicine (ICPM) defined by WHO. According to the OPS codes version 2006–2010, a systemic thrombolysis has the OPS code 8-020.8. Because there is no specification for the specific indication, we restricted our analysis to neurology departments in order to exclude the possible inclusion of thrombolysis due to an indication other than acute ischemic stroke (e.g., myocardial infarction or thromboembolic event). Given the fact that almost 70% of all ischemic stroke patients were treated by a neurology department in Germany in 2010 [36], the restriction of our study to neurology departments limits the number of analyzed ischemic stroke cases. Nevertheless, our analysis still accounts for the majority of stroke patients.

Along with the OPS code for systemic thrombolysis, we also identified the documented OPS codes 8-981 and 8-98b. These define departments as being specialized in the treatment of acute stroke patients (stroke unit). The requirements for the use of the codes are the existence of a specialized team for treating acute stroke as well as the utilization of monitoring devices, the existence of a facility for computer tomography imaging, and structural conditions that allow for the performance of systemic thrombolysis. The thrombolysis rate (TR) was calculated by dividing the number of coded systemic thrombolysis procedures by the number of coded ischemic stroke patients per year. For the year 2006, it was possible to analyze data from 286 different neurology departments that treated more than 113,700 ischemic stroke patients. To evaluate the influence of the guideline update in 2009, we measured the thrombolysis rate change between 2008 and 2010. To this end, the data from 367 departments in 2008 was matched with the data published by the hospitals for the year 2010. As a matching variable, we used the institutional code, an unambiguous identification number, and the hospital’s postal code. Hospitals that could not be matched by postal code and institutional code were matched by their name and postal code. Of the 367 hospitals that treated ischemic stroke patients in a neurology department in 2008, 348 could be matched. Overall, 19 hospitals in 2008 and 29 hospitals in 2010 that treated ischemic stroke patients could not be matched by this procedure.

According to Lehrman et al., organizational characteristics influence a hospital’s clinical processes and measures of care [25]. For this reason, we included hospital organizational and departmental-level parameters in our study. On the hospital level, these parameters are ownership, hospital size (measured by the total number of beds), teaching status, and location. A hospital’s location is defined by its urbanization grade in accordance with the definition of the German Federal Statistics Bureau (i.e., urban, semi-urban, and rural). We therefore matched the hospital data with a list of municipalities from the year 2010 (as published by the Federal Statistics Bureau). As an explanatory variable at the departmental level, we included in the models the existence of a stroke unit as defined by the OPS codes 8-981 and 8-98b as well as the number of treated ischemic stroke patients.

To identify possible organizational factors influencing the performance of systemic thrombolysis on acute ischemic stroke patients, we calculated a multiple logistic regression model for the year 2006. The dependent variable here was the performance of systemic thrombolysis as early as 2006, categorized as a dichotomous variable with the specification ‘performance of systemic thrombolysis: “yes” or “no”. Explanatory variables were ownership, number of beds, teaching status, existence of a stroke unit, and the number of treated ischemic stroke patients. The logistic regression analysis was based on 280 neurology departments, as six hospitals were excluded from this analysis because of a lack of data related to ownership.

The Wilcoxon signed-rank test was performed to compare the thrombolysis rate in 2008 and 2010 in order to examine the rate before and after the guideline update in 2009.

We calculated a multiple linear regression model to determine whether there were factors at the organizational level that enabled hospital departments to implement the guideline update in 2009. The dependent variable was the systemic TR change between 2008 and 2010, measured as the difference between the TR in 2008 and 2010 for all hospitals that performed thrombolysis in 2008. As with aggregated data, the depth of implementation cannot be measured directly; the TR change functions as a surrogate indicator. As explanatory variables, parameters on the hospital and department level (i.e., ownership, number of beds, location, degree of urbanization, teaching status, existence of a stroke unit, and the number of treated ischemic stroke patients) were included in Model 1. In Model 2, we took the TR in 2008 into account in order to adjust for the depth of implementation that had already been achieved.

Data analysis and statistical calculations were performed using STATA 12.

## Results

**Figure 1:**
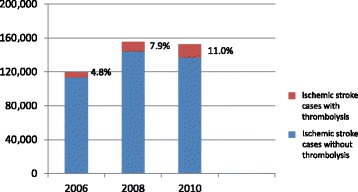
**Ischemic stroke patients treated in a neurology department with and without thrombolysis.**

The number of ischemic stroke patients treated by a neurology department increased from approximately 113,700 in 2006 to around 144,000 in 2008 but then decreased to roughly 136,800 in 2010. The percentage of ischemic stroke patients receiving thrombolysis in a neurology department continuously increased from approximately 4.8% in 2006 to approximately 11.0% in 2010 (mean departmental thrombolysis rates: approximately 4.6% and 9.2%, respectively) (Figure 1). With 61.5% of neurology departments (176 out of 286) performing thrombolysis in 2006, we can see that the majority were able to offer a guideline-congruent therapy. However, there were also a significant number of departments that did not implement this therapeutic option into their treatment routine. In 2010, the share of neurology departments that treated acute ischemic stroke patients and also performed thrombolysis increased to 77.5%.

In 2006, 61.5% of the neurology departments coded the utilization of systemic thrombolysis. We calculated a multiple logistic model to determine whether hospital/department characteristics influenced a neurology department’s implementation of the guideline, and of the only approved curative and causal therapy (i.e., systemic thrombolysis), as early as 2006. The logistic regression model (Table 1) shows the positive influence of the existence of a stroke unit. For the hospital’s size, measured by the number of hospital beds, we found a negative effect for hospitals with between 500 and 1,000 beds. None of the other variables displayed any significant relationships.

**Table 1:** **Logistic regression model: performance of systemic thrombolysis in 2006 (thrombolysis yes or no)**

**Explanatory variable**	**B**	**(95% CI)**	**Standard error of B**	***p*** **value**
Constant	−0.142	(−1.838 to 1.354)	0.763	0.853
Ownership (ref. public)				
Private non-profit	−0.737	(−1.508 to 0.034)	0.394	0.061
Private	−0.750	(−1.683 to 0.182)	0.476	0.115
Location (ref. urban)				
Semi-urban	−0.387	(−1.224 to 0.449)	0.427	0.364
Rural	−0.904	(−2.154 to 0.346)	0.638	0.156
Teaching status (ref. teaching hospital)				
University hospital	0.671	(−0.179 to 1.521)	0.434	0.122
Non-teaching hospital	−0.165	(−2.203 to 1.872)	1.039	0.874
Stroke unit (ref. No)				
Yes	2.876	(2.167 to 3.585)	0.362	0.000
Number of beds (ref. <100)				
100–199	−0.528	(−2.007 to 0.952)	0.755	0.485
200–499	−1.206	(−2.607 to 0.197)	0.715	0.092
500–999	−1.754	(−3.346 to -0.139)	0.818	0.033
>1,000	−1.810	(−3.758 to 0.138)	0.994	0.069
Number of stroke patients in 2006	0.001	(−0.000 to 0.002)	0.000	0.080
Pseudo R2 = 0.31, *N* = 280				

After matching the data from 2008 and 2010, we found that 7.8% of the neurology departments that did not perform thrombolysis in 2008 did treat ischemic stroke patients with systemic thrombolysis in 2010. Overall, 51.1% of the neurology departments increased their thrombolysis rate between 2008 and 2010. By contrast, 22.4% of the neurology departments decreased their thrombolysis rate, and 18.4% of the neurology departments did not perform systemic thrombolysis in acute ischemic stroke patients either in 2008 or in 2010.

**Figure 2:**
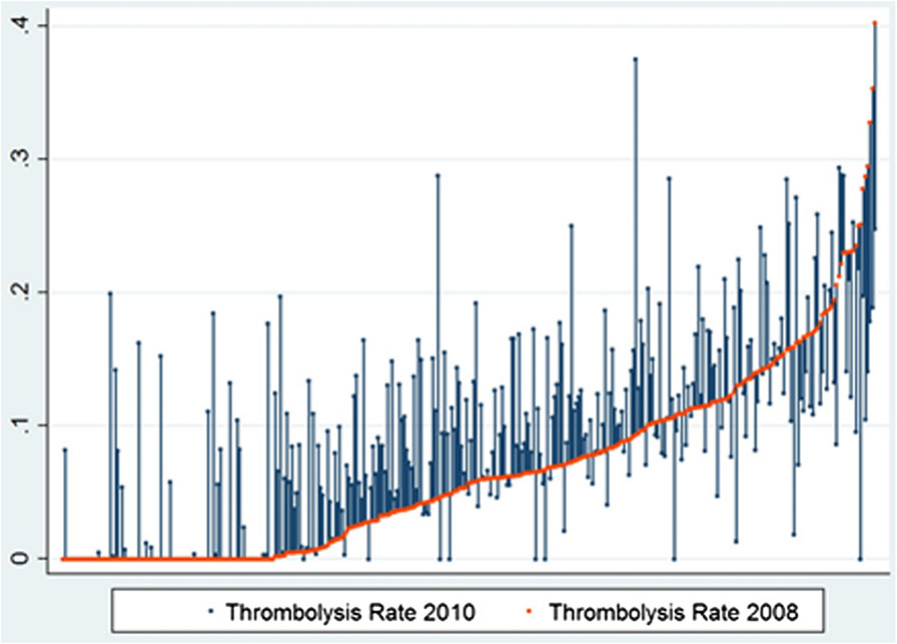
**Thrombolysis rates in 2010 and 2008:** **each line represents one neurology department.**

A thrombolysis-rate ranging from 0% up to 35.7% of ischemic stroke patients in 2010 constitutes a substantial variation between neurology departments (Figure 2).

Each line represents one neurology department. Changes in the thrombolysis rate are illustrated by the spikes grounding on the red line, showing upward (TR up) or downward (TR down) movements. The Wilcoxon signed-rank test showed a significant increase (2.5%) in the thrombolysis rate between 2008 and 2010.

Calculating a linear regression model on the TR change between 2008 and 2010, we did not find any significant factors at the organizational level that might have influenced the TR change. (Table 2—Model 1). The purpose of adding the 2008 TR into the model was to measure the effect that the depth of implementation already achieved had on the TR change after the guideline update in 2009 (Table 2—Model 2). The level of the thrombolysis rate in 2008 had a significant influence on the TR change between 2008 and 2010. For example, we were able to show that a high TR in 2008 was associated with a TR decrease in 2010. This effect was significant for TRs of over ten percentage points in 2008.

**Table 2:** **Linear regression on the thrombolysis rate change between 2008 and 2010 for all neurology departments performing thrombolysis**

**Model 1**					**Model 2**			
**Explanatory variable**	**B**	**(95% CI)**	**Standard error of B**	***p*** **value**	**B**	**(95% CI)**	**Standard error of B**	***p*** **value**
Constant	0.069	(−0.065 to 0.203)	0.068	0.312	0.0715	(−0.044 to 0.187)	0.058	0.225
Ownership (ref. public)								
Private non-profit	0.004	(−0.018 to 0.025)	0.011	0.740	0.0018	(−0.017 to 0.020)	0.009	0.851
Private	0.000	(−0.024 to 0.024)	0.012	0.981	−0.0070	(−0.027 to 0.013)	0.010	0.500
Location (ref. urban)								
Semi-urban	0.004	(−0.019 to 0.027)	0.011	0.731	0.0040	(−0.016 to 0.024)	0.010	0.683
Rural	−0.002	(−0.035 to 0.031)	0.017	0.907	−0.0024	(−0.031 to 0.026)	0.015	0.872
Teaching status (ref. university hospital)								
Teaching hospital	0.009	(−0.026 to 0.043)	0.017	0.614	−0.0102	(−0.040 to 0.020)	0.015	0.504
Non-teaching hospital	0.002	(−0.041 to 0.044)	0.021	0.938	−0.0210	(−0.055 to 0.011)	0.017	0.259
Stroke unit (ref. no stroke unit)								
Stroke unit only in 2010	−0.046	(−0.139 to 0.047)	0.047	0.333	−0.0037	(−0.085 to 0.078)	0.041	0.929
Stroke unit only in 2008	−0.045	(−0.172 to 0.082)	0.064	0.486	−0.0210	(−0.132 to 0.090)	0.056	0.709
Stroke unit in 2008 and 2010	−0.029	(−0.110 to 0.052)	0.041	0.477	−0.0001	(−0.070 to 0.070)	0.036	0.997
Number of beds (ref. <100)								
100–199	−0.063	(−0.169 to 0.042)	0.054	0.239	−0.0388	(−0.130 to 0.052)	0.046	0.404
200–499	−0.027	(−0.126 to 0.071)	0.050	0.584	−0.0077	(−0.093 to 0.078)	0.043	0.858
500–999	−0.036	(−0.136 to 0.064)	0.051	0.481	−0.0130	(−0.099 to 0.074)	0.044	0.769
>1,000	−0.037	(−0.140 to 0.067)	0.053	0.487	−0.0109	(−0101 to 0.079)	0.046	0.812
Number of stroke patients in 2008	0.000	(−0.000 to 0.000)	0.000	0.096	0.000	(−0.000 to 0.000)	0.000	0.233
Thrombolysis rate 2008 (ref.: <0.5)								
≥0.05 and <0.10					−0.0174	(−0.037 to 0.002)	0.010	0.076
≥0.10 and <0.20					−0.0462	(−0.065 to −0.027)	0.010	0.000
≥0.20					−0.1500	(−0.183 to −0.117)	0.017	0.000
Adj. R2 = −0.02, *N* = 256					Adj. R2: 0.25, *N* = 256			

As regards the implementation of thrombolysis as a treatment for acute ischemic stroke between 2008 and 2010, the measured factors on the organizational level were not significant—apart from the fact that none of the 27 neurology departments that began performing systemic thrombolysis between 2008 and 2010 were located in a rural setting.

## Discussion

The implementation of systemic thrombolysis in acute ischemic stroke care can reduce the burden of stroke [38]. Although systemic thrombolysis remains the only approved curative and causal treatment [5] to date, many countries around the world still struggle with a thrombolysis rate below the possible optimum [32,39], as well as with the fact that hospitals do treat acute stroke patients but not by performing systemic thrombolysis [35]. It is therefore necessary to identify possible barriers on the organizational level that prevent the implementation of thrombolysis in acute ischemic stroke care. Our analyses show that an increasing number of neurology departments performed systemic thrombolysis between 2006 and 2010.

### Influencing factors in 2006

We calculated a model for 2006 in order to answer the question as to which organizational factors influenced the implementation of systemic thrombolysis in that year. At the organizational level, we identified the existence of a stroke unit (+) and hospital size (between 500 and 1,000 beds) (−) as predicting factors. By contrast, a study from the Netherlands found that systemic thrombolysis was used far more in an academic setting [30]. We did not observe a difference between teaching and non-teaching hospitals in our study, which indicates that hospitals in both non-academic and academic settings will implement to a similar degree a fundamental novel therapy, such as thrombolysis for acute cerebral ischemia. The same applies to a hospital’s location: hospitals in urban regions were no more likely to perform systemic thrombolysis than hospitals in rural regions. These findings correspond with the results from an Australian study [32]. The question as to why hospitals with between 500 and 1,000 beds were less likely to have implemented systemic thrombolysis as early as 2006 needs to be addressed through further research.

### The TR change in 2010

It cannot be said whether the TR increase between 2008 and 2010 can be explained by the guideline update in 2009 (resulting in an extended time window for performing systemic thrombolysis) or if it was instead part of an ongoing process of implementing systemic thrombolysis for the treatment of acute ischemic stroke patients. It is noteworthy that the time frame between the guideline update and our assessment was not very long. However, the extension of the time window in which thrombolysis can be performed did not constitute a completely new approach but rather an extension of an already established treatment modality. In Sweden, thrombolysis within the extended time frame was implemented just a few months after new recommendations and study protocols were published [40]. The Riks-Stroke study attributed the TR increase to the prolonged therapeutic time interval, as the TR increase within 3 h after the onset of symptoms had leveled off since the end of 2008 in Sweden [40]. Similar results were obtained in the SITS-ISTR study, where a rapid implementation process was reported, accompanied by a simultaneous increase in ischemic stroke patients treated with systemic thrombolysis within the 3-h time frame [17].

We did not find any factors at the organizational level that influenced the TR change between 2008 and 2010. These results are in line with the findings of the Riks-Stroke study, which found that non-university hospitals were just as fast as university hospitals in implementing the new recommendations [40].

The negative influence of a high TR in 2008 on the TR change in 2010 implies that it is difficult to continuously maintain a very high TR. It seems that in a system where thrombolysis is already very well established, it is not possible for the TR to rise beyond a certain level. Moreover, even though the time window for applying thrombolysis has been extended, patients should nevertheless still be treated as quickly as possible after symptom onset, as the chances of recovery decrease as the interval between symptom onset and treatment gets longer [12]. Stroke-experienced environments with well-established processes and short delays between symptom onset and hospital admission are thus likely to benefit less from the extended time window. Nevertheless, further research into this issue is warranted.

### The organizational level and beyond

Our analysis suggests that systemic thrombolysis was continuously implemented between 2006 and 2010. Whether or not a neurology department performs thrombolysis is a decision to be made by each hospital that treats ischemic stroke patients. If one considers innovation as a continuous process between non-use and committed use [22], the thrombolysis rate would stand for the implementation level of this innovative treatment. Increasing the TR is a challenge that involves several levels [41]. According to Chaudoir et al. [23], there are five different levels (the structural, organizational, patient, provider, and innovation levels) that influence the implementation outcome. Our analysis is based on the structural level. Klein and Sorra state that effective implementation is influenced by the implementation climate and the innovation-values fit [22]. This, in turn, is influenced by the structural level. If the implementation climate, which exists at the organizational level [42], plays a significant role, should be analyzed in future research, along with the organizational readiness to change [43]. Organizational readiness consists of the organization members’ knowledge and willingness to make a change, and it also includes the organizational resources available to implement the change [44]. In our study, we estimated the effects of ownership, hospital size (number of beds), location, teaching status, existence of a stroke unit, and number of treated ischemic stroke patients as organizational-level factors that might potentially influence the depth of implementation of systemic thrombolysis for ischemic stroke patients. We therefore estimated the effect of hospital/department level issues on implementation behavior with regard to systemic thrombolysis. Our analysis comprises only structural organizational effects. Along with structural organizational factors that influence the implementation routine, organizational aspects such as leadership and social interaction have also been identified as crucial elements in the implementation of thrombolytic therapies for acute ischemic stroke patients [45]. Making a decision to change behavior and perform a guideline-congruent treatment is, at least in part, the responsibility of individual health professionals [46]. Here—i.e., in the realm of social cognitive theories—there is a need for more studies that might answer the question regarding the nature of the interaction between innovation implementation and the individual level [47]. Barriers that hinder the use of systemic thrombolysis to treat acute stroke patients can be found on both the pre-admission (in the form of patient-related and paramedic-related barriers) and the post-admission level [48].

Because systemic thrombolysis can only be performed if the ischemic stroke patient arrives at the hospital within the therapeutic time frame, action has been taken in Germany in recent years to inform the public about the relevant symptoms of a stroke and how important it is to get to the hospital immediately. Although a German study suggests that 54% of German stroke patients reach a hospital within 3 h after symptom onset [49], the published overall TR remained at 8.9% in 2010 [3]. This suggest that efforts to achieve a significant improvement in stroke treatment must also focus on hospitals and the things that prevent the application of systemic thrombolysis, even if the patients arrive at the hospital within the therapeutic time frame of 4.5 h after the onset of symptoms. Similar results have been found for the UK, where over 28% of acute stroke patients were present in a hospital on time, based on a 3 h rule [33].

### The international perspective

Although scientific results are distributed internationally, new guidelines and recommendations have to be implemented in each country and health-care system—and in every hospital. Because the implementation process is influenced by many factors [23], the question also has to be raised regarding the extent to which these factors are influenced by specific national characteristics. Several studies have shown the effect the organizational level has on the implementation of guidelines, with similar results having been obtained across different national settings [24-32]. With this in mind, we focused on organizational level parameters that can be compared on an international scale: ownership, hospital location, hospital size, teaching status, and the existence of a stroke unit. An international group of researchers identified the TR as a potential performance measure for the comparison of different organizational structures or countries, although population bias has to be accounted for as well [50]. To reduce the population effect to a minimum, we focused in our study on the implementation of systemic thrombolysis and on the TR change as parameters that are generally not influenced by the patient level. To our knowledge, this is the first study to analyze the effect of these parameters on organizational behavior in terms of implementing thrombolysis to treat acute stroke patients (based on nationwide data).

### Limitations

Our study has some limitations. The analysis is based on administrative data provided by hospitals. Although all licensed hospitals are required to publish a structured quality report, not all hospitals comply with this legal obligation. According to our estimates, less than 5% of all German hospitals do not release their data to the public. The data basis is routinely collected for claim and administrative purposes, which explains why only documented medical procedures are accounted for. Because we did not have access to clinical data, our analyses were restricted to the organizational level. We were therefore not able to consider the patient level. Information about the patient level is essential for comparing the TR between hospitals, or even between countries, as an absolute measure, as population bias resulting from national characteristics can occur [50]. Hospital density or national stroke awareness campaigns can have an impact on the pre-admission level, for example. The first year data was available on the hospital level in Germany was 2006. With systemic thrombolysis having been licensed in Germany as early as 2000, an analysis of implementation at an earlier date would have been preferable.

## Conclusion

Who does it first? Our data suggests that the implementation of thrombolysis in the treatment regime for acute ischemic stroke patients is mainly influenced by the existence of a stroke unit (+), and in part, by organizational size (−) as well. The results indicate that teaching status, ownership, and location did not influence the implementation of systemic thrombolysis. This result can encourage different types of hospitals that treat ischemic stroke patients to establish stroke units and incorporate systemic thrombolysis into their daily routines, since according to our data and the measured characteristics, there seem to be no other structural barriers at the organizational level. On the other hand, further research must be done in order to gain greater insight into the organizational climate, which influences organizational responsiveness.
